# Epstein-Barr Virus-Associated T and NK-Cell Lymphoproliferative Diseases

**DOI:** 10.3389/fped.2019.00071

**Published:** 2019-03-15

**Authors:** Wook Youn Kim, Ivonne A. Montes-Mojarro, Falko Fend, Leticia Quintanilla-Martinez

**Affiliations:** ^1^Institute of Pathology and Neuropathology and Comprehensive Cancer Center Tübingen, University Hospital Tübingen, Eberhard-Karls-University, Tübingen, Germany; ^2^Department of Pathology, Konkuk University School of Medicine, Seoul, South Korea

**Keywords:** EBV, lymphoproliferations, hemophagocytic lymphohistiocytosis, chronic active EBV infection, systemic EBV positive T-cell lymphoma, aggressive NK-cell leukemia, extranodal NK/T-cell lymphoma, primary EBV nodal T and NK-cell lymphoma

## Abstract

EBV-associated T and NK-cell lymphoproliferative diseases (EBV-T/NK LPDs) are characterized by the transformation and proliferation of EBV-infected T or NK cells. The 2016 revised World Health Organization classification recognizes the following EBV-positive lymphoproliferative disorders (LPD): chronic active EBV infection (CAEBV) of T- and NK-cell type (cutaneous and systemic forms), systemic EBV-positive T-cell lymphoma of childhood, aggressive NK-cell leukemia, extranodal NK/T-cell lymphoma, nasal type, and the new provisional entity primary EBV-positive nodal T/NK-cell lymphoma. EBV-associated hemophagocytic lymphohistiocytosis (HLH), although not included in the WHO classification because it is a reactive, inflammatory disease, is included in this review because it can be life-threatening and may have overlapping features with other EBV+ T/NK LPDs. EBV+ T/NK LPDs are rare diseases difficult to diagnose and manage properly, because some LPDs have unusual presentations, and discrepancies between clinical and histological findings might be encountered. Furthermore, EBV+ T/NK disorders share some clinico-pathological features, and may evolve into other categories during the clinical course, including malignant transformation of CAEBV. Here, we review the EBV+ T/NK LPDs in terms of their definitions, clinical features, histology, immunophenotype, molecular findings, and pathogenesis. This review aims to increase our understanding and awareness of the differential diagnosis among the different EBV+ T/NK LPDs. New insights into the genetic characteristics of these disorders will also be discussed.

## Introduction

Epstein-Barr virus (EBV) is a gamma herpesvirus with a double-stranded DNA genome in core. EBV has a tropism for B cells but can infect various types of human cells including T cells, NK cells and even epithelial cells (1). EBV causes chronic latent infection with lifelong persistence in about 95% of the world population (2). Memory B cells are important as the main reservoir of EBV during the latency period. The impairment of balance between host immune response and EBV virus can lead to various EBV-associated lymphoproliferative disorders (LPDs) of B, T, or NK cells. EBV-associated LPDs can be categorized into B or T/NK-cell types based on which cells are infected by the virus.

EBV-associated T and NK-cell LPDs are characterized by the transformation and proliferation of EBV-infected T and NK-lymphocytes that usually carry an EBV-latency type 2. These disorders occur commonly in Asians and Native Americans from Central and South America (3). EBV-associated T and NK-cell LPDs represent a broad spectrum of diseases encompassing various reactive and malignant disorders (4). They are classified into 6 categories, consisting of EBV-associated hemophagocytic lymphohistiocytosis (HLH), chronic active EBV infection (CAEBV) of T- and NK-cell type, systemic EBV-positive T-cell lymphoma of childhood, aggressive NK-cell leukemia, extranodal NK/T-cell lymphoma, nasal type, and primary EBV-positive nodal T/ NK-cell lymphoma, the latter incorporated as a new provisional subgroup within peripheral T-cell lymphoma, not otherwise specified (Table 1). The first three disorders are prevalent in the pediatric and adolescent population, whereas the last three groups usually affect adults (3). Systemic EBV-positive T-cell lymphoma of childhood (previously LPD), aggressive NK-cell leukemia, extranodal NK/T-cell lymphoma, nasal type, and primary EBV-positive nodal T/NK-cell lymphoma are considered malignant proliferations in the 2016 revised WHO classification (3). CAEBV of T- and NK-cell type represents a reactive process of EBV-associated T and NK-cell LPDs with potential to progress into a malignant disorder. CAEBV is divided into one systemic and two cutaneous forms; hydroa vacciniforme (HV)-like LPD (previously lymphoma) and severe mosquito bite allergy. EBV-associated HLH is a clinicopathological syndrome complicated by abnormal hyperinflammatory immune response and is also considered a reactive disorder.

**Table 1:** EBV-associated T and NK- cell lymphoproliferative diseases.

**Disease entities**
EBV-positive hemophagocytic lymphohistocytosis
Chronic active EBV infection of T- and NK-cell type
Systemic form
Cutaneous form
Hydroa vacciniforme-like lymphoproliferative disease
Severe mosquito bite allergy
Systemic EBV-positive T-cell lymphoma of childhood
Aggressive NK-cell leukemia
Extranodal NK/T-cell lymphoma, nasal type
Primary EBV-positive nodal T and NK-cell lymphoma*

* *Considered a provisional entity within peripheral T-cell lymphoma, not otherwise specified*.

This review aims to describe the clinicopathological features of various EBV-associated T and NK-cell LPDs including reactive lymphoproliferations as well as overt lymphomas. The main features are summarized in Table 2.

**Table 2:** Summary of pathological features of EBV-associated T and NK- cell lymphoproliferative diseases.

	**Clinical features**	**Histological features**	**Immunophenotypic features**	**Lineage and Clonality**
EBV-associated HLH	High fever and splenomegaly Cytopenia and liver dysfunction Serological test or the detection of EBV DNA or RNA from the tissues Exclusion of other EBV-associated T/NK-LPDs	Hemophagocytosis by activated histiocytes in BM, spleen, or LNs Presence of relatively small numbers of EBV+ T cells	Predominantly cytotoxic CD8+ T cells	T cell (80%) NK cell (20%) Monoclonal TCR (50%)
CAEBV, systemic	Persistent IM-like illness >3 months in duration High fever, hepatosplenomegaly, HV-like eruptions, hypersensitivity to mosquito bite, uveitis, diarrhea, and lymphadenopathies Cytopenia and liver dysfunction Increased EBV DNA (>102.5 copies/mg) in peripheral blood or demonstration of EBV RNA or viral protein in affected tissues	Nonspecific inflammatory changes with no histological evidence of malignant lymphoproliferations	CD4>>CD8> γδ T cells CD56+ (41%)	T cell (59%) NK cell (41%) Monoclonal TCR (50%) Monoclonal EBV (84%)
Hydroa vacciniforme-like LPD	Cutaneous form of CAEBV Recurrent vesiculopapular eruptions usually in sun-exposed skin area Indolent, self-limited clinical course with a risk to progress to other EBV-associated T/NK-LPDs	Intraepidermal spongiotic vesicles Lymphoid infiltrates with angiocentric and periadnexal involvement Small lymphocytes with no or mild atypia	Predominantly cytotoxic CD8+ T cells CD56+ (30%)	T cell (70%) NK cell (30%) Monoclonal TCR Monoclonal EBV
Severe mosquito bite allergy	Cutaneous form of CAEBV Exaggerated hypersensitivity reaction to mosquito bites (erythema, bullae, ulcers, scarring, high fever, lymphadenopathy, liver abnormalities, and hepatosplenomegaly) Prolonged clinical course with a risk to progress to other EBV-associated T/NK-LPDs	Epidermal necrosis, ulcer and bullae Polymorphous infiltration of small lymphocytes, large atypical cells, and other reactive inflammatory cells including histiocytes and eosinophils	CD3ε+, CD56+ NK cells	NK cell Polyclonal TCR Monoclonal EBV
Systemic EBV+ T-cell lymphoma of childhood	High fever, hepatosplenomegaly, pancytopenia, and coagulopathy, and abnormal liver function Monoclonal proliferation of EBV-positive T cells in tissues or peripheral blood Occurs shortly after acute primary EBV infection in previously healthy children or in the setting of CAEBV Fulminant clinical course that resulted in death within days to weeks	Increased infiltration of small lymphoid cells with histiocytic hyperplasia and striking hemophagocytosis in BM, spleen, and liver Small lymphocytes with no or minimal atypia	Predominantly CD8+ cytotoxic T cells CD2+, CD3+	T cell Monoclonal TCR
Aggressive NK-cell leukemia	High fever, general malaise, hepatosplenomegaly, hepatic failure, and pancytopenia Systemic neoplastic proliferations of NK cells in peripheral blood and bone marrow Fulminant clinical course Presence of EBV-negative subset (<15%)	Varying degrees of leukemic cell infiltration in BM, LN, liver and spleen (sometimes focal or subtle) A broad cytological spectrum ranging from normal large granular lymphocytes to atypical pleomorphic lymphocytes	CD3ε+, CD56+ NK cells CD2+, FASL+ surface CD3-, CD5- CD16+ (75%)	NK cell Polyclonal TCR
Extranodal NK/T-cell lymphoma, nasal type	EBV-positive aggressive lymphoma Nasal type (70–80%): occurs in nasal and nasopharyngeal area, relatively less aggressive disease Extranasal type (20–30%): in skin, GI tract, and testis, aggressive disease Extensive ulceration and necrosis in mucosal sites	Diffuse infiltration of atypical lymphoid cells with angiocentricity and angiodestruction Frequent coagulative necrosis Broad cytological spectrum Variable amounts of inflammatory cells	Mostly CD3ε+, CD56+ NK cells (CD25+, FAS+, FASL+, HLA-DR+, surface CD3-, CD4- CD5-) Occasionally CD3ε+, CD56- cytotoxic T cells (CD8+, CD5+,TCR γδ or αβ+)	NK cell (80–85%) T cell (15–20%) Monoclonal TCR (10–40%)
Primary EBV+ nodal T/NK-cell lymphoma	A rare type of EBV+ PTCL with primary nodal presentation Generalized lymphadenopathy Limited extranodal lesions without nasal involvement	Relatively monomorphic proliferation of large atypical cells with centroblastic feature or diffuse proliferation of pleomorphic cells composed of small, medium, to large atypical cells	Predominantly CD8+ cytotoxic T cells, γδ T cells CD56+ (7.5–15%), CD4+ (15–20%)	Mostly T cell, rarely NK cell Monoclonal TCR

*EBV, Epstein barr virus; HLH, hemophagocytic lymphohistiocytosis; CAEBV, chronic active EBV infection; NK, natural killer; BM, bone marrow; LN, lymph nodes*.

*TCR, T-cell receptors; GI tract, gastrointestinal tract; PTCL, peripheral T-cell lymphoma; LPDs, lymphoproliferative disorders; IM, infectious mononucleosis*.

## EBV-Associated Hemophagocytic Lymphohistiocytosis

HLH is a life-threatening inflammatory disease characterized by uncontrolled and overwhelming activation of the immune system. HLH is a clinical syndrome that can be diagnosed when the patient meets diagnostic criteria based on the following findings: (1) clinical features such as fever and splenomegaly; (2) laboratory abnormalities including cytopenias, hyperferritinemia and liver dysfunction; and (3) pathological findings showing hemophagocytosis in bone marrow (BM), spleen, or lymph nodes (LN) (4, 5). HLH can be divided into primary and secondary forms according to underlying causes. The primary or familial type is an inherited disorder with an autosomal recessive inheritance of mutations affecting the cytotoxic function of T and NK cells, and the secondary type corresponds to an acquired type associated with various conditions including infections, autoimmune disorders, and malignancy (6, 7). However, the clinical distinction between primary and secondary HLH may be ambiguous, because patients with HLH-associated gene defects frequently have an infectious event triggering the clinical symptoms (8). Furthermore, advanced molecular techniques including whole-exom sequencing can identify underlying genetic abnormalities that have not been detected by conventional clinical testing in patients with presumed secondary HLH (9). Recent whole-genome sequencing analysis identified several types of genetic defects related with immune dysfunction, including potentially novel ones, in 58% of patients with HLH (9). Therefore, systematic and comprehensive investigations are essential for all patients with newly diagnosed HLH.

Viral infections are the most common cause of secondary HLH, and EBV is the most frequently HLH-associated virus (5). In a nationwide survey performed in Japan, EBV-associated HLH accounted for about 30% of HLH, followed by other infection- or lymphoma-associated HLH (5). EBV infection was reported to be about 75% in patients with HLH in a retrospective study done in China (10). HLH can also occur in the clinical course of other EBV-associated T and NK-cell LPDs including CAEBV of T/NK cell type or systemic EBV-positive T-cell lymphoma of childhood. An independent diagnosis of EBV-associated HLH, as a separate entity, can be made when other EBV-associated LPDs are excluded in the differential diagnosis. EBV-associated HLH is typically associated with EBV infection of T or NK cells rather than B cells.

Among several predisposing genetic conditions, X-linked lymphoproliferative disease (XLP) is often associated with EBV-associated HLH. Especially, HLH arising in patients with XLP type 1 is nearly exclusively linked to EBV (11). In patients with XLP type 2, HLH is commonly associated with EBV, but also arises in response to other infectious agents besides EBV or without an identifiable infectious cause (11). EBV-associated HLH occurs predominantly in children and adolescents. The majority of cases have been reported in East Asians (5, 12). Differences in geographic distribution indicates that there may be some genetic predisposition in the pathogenesis of EBV-associated HLH (13).

### Clinical Features

EBV-associated HLH typically presents with continuous high-grade fever and splenomegaly. Other findings including lymphadenopathy, jaundice, edema, and skin rash may also be present. Laboratory tests show cytopenias affecting more than two lineages in the peripheral blood (PB), abnormalities in liver function tests (e.g., hypertriglyceridemia, hypofibrinogenemia, elevated serum transaminases, hyperbilirubinemia, prolonged prothrombin time, and prolonged partial thromboplastin time), hyperferritinemia, cerebral spinal fluid (CSF) pleocytosis, hyponatremia, and hypoproteinemia (6). Among these, cytopenia, hypertriglyceridemia, hypofibrinogenemia, and hyperferritinemia are included in the diagnostic criteria of HLH (Table 3) (14). EBV-HLH tends to show these clinical findings in a more rapid and severe fashion, compared to other HLHs (12). However, clinical manifestations may be highly variable and some patients can have nonspecific presentations by unusual organ involvement, including intestinal perforation, cutaneous lesions, pulmonary infiltrates, and central nervous system (CNS) disease (12, 13, 15). Therefore, clinical suspicion is important for the appropriate diagnosis and treatment of EBV-associated HLH.

**Table 3:** Diagnostic guidelines for HLH used in the HLH-2004 trial.

The diagnosis of HLH can be established if one of either 1 or 2 below is fulfilled. 1. A molecular diagnosis consistent with HLH. 2. Diagnostic criteria for HLH fulfilled (at least 5 out of the 8 criteria below) 1) Fever ≥38.5°C 2) Splenomegaly 3) Cytopenia involving ≥2 cell lines
Hemoglobin <90 g/L (in infants <4 weeks: hemoglobin <100 g/L)
Platelets <100 × 109/L
Neutrophils <1.0 × 109/L
4) Hypertriglyceridemia (fasting, ≥265 mg/dL) or hypofibrinogenemia (≤1.5g/L) 5) Hemophagocytosis in bone marrow, spleen, or lymph nodes 6) Low or absent natural killer cell activity 7) Serum ferritin > 500μg/L 8) Elevated CD25 (soluble IL-2 receptor) levels (>2400 U/mL)

EBV-associated HLH induces hypercytokinemia resulting from abnormal hyperactivation of the immune system. Various cytokines including IFN-γ, TNF, sIL-2R, IL-6, IL-10, and IL-18 are secreted by activated macrophages and T-lymphocytes (12). They have been used as biological indicators reflecting the severity of HLH. The level of sIL-2 receptor (sCD25) is used as a diagnostic marker. It is crucial to detect EBV infection in the patient for diagnosing EBV-associated HLH. The presence of EBV can be determined by serological tests or by detection of EBV DNA or RNA from PB or any tissue. Viral load can be estimated by measuring EBV copy numbers using the real time PCR assay, which correlates better with clinical severity than serology (12). The clinical course of EBV-associated HLH varies from mild to severe or fatal.

### Morphology and Immunophenotypical Findings

Histologically, activated macrophages engulfing RBCs, leukocytes, platelets, and their precursor cells are scattered in the sinusoids of BM, spleen, liver, and LN, but are not required for diagnosis. EBV-infected T cells are also found with hemophagocytic histiocytes and show a cytotoxic phenotype with expression of CD8 and granzyme B in the majority of cases (4). The characteristics of infiltrating T cells are different between EBV-associated HLH and other EBV-associated T and NK-cell LPDs. Generally, EBV+ T cells frequently show a cytotoxic phenotype with CD8 expression in EBV-associated HLH and systemic EBV-positive T-cell lymphoma, whereas CD4+ cells or NK cells are predominantly infected by EBV in other EBV-associated T and NK-cell LPDs. In addition, the EBV+ cells are present in relatively small amounts in EBV-associated HLH, compared to other LPDs. *In situ* hybridization (ISH) with the EBV-encoded small RNA (EBER) is used to detect EBV-infected cells. Double staining with EBER ISH and CD20, CD3, or CD56 can be done to identify which cells are infected by EBV. HLH induced by EBV-infected NK cells has been reported to occur uncommonly, accounting for 20% in a previous report (4, 16).

### Pathogenesis and Molecular Features

The precise mechanism on how T or NK cells lacking CD21, the primary receptor for EBV, are infected by EBV in EBV-associated HLH is still unknown. A previous report showed that CD21 is synaptically transferred to NK cells through conjugation to CD21+, EBV-infected B cells, thereby allowing EBV binding to NK cells (16, 17). T-cell receptor (TCR) gene rearrangement can be detected in about half of cases with EBV-associated HLH using conventional method (18). Furthermore, with the introduction of Biomed-2 multiplex PCR, the detection rate of T-cell clonality is notably increasing in EBV-associated HLH. It has been suggested that changes in T cell clonality pattern (monoclonal to polyclonal) could be helpful to predict the therapeutic response of patients (18).

Many predisposing genetic conditions of HLH are characterized by impaired cytotoxicity of cytotoxic T or NK cells. Familial HLH 2, 3, 4, and 5 are caused by mutations in *PRF1, UNC13D, STX11*, and *STXBP2*, respectively (19–22). In patients with these mutations, cytotoxic cells demonstrate functional impairment in the degranulation process including cytotoxic granule docking, priming, and fusion with the plasma membrane, when encountering susceptible target cells infected with virus (11, 23). As a result, target cells are not eliminated by cytotoxic lymphocytes, and persistently activate immune cells, ultimately leading to a hyperinflammatory HLH (11). Among these mutations, *PRF1* mutation induces total deficiency of functional perforin, which results in defective cytotoxicity of cytotoxic T or NK cells (24). The pathogenetic mechanism of XLP-associated HLH is more complicated. Patients with XLP type 1 harbor mutations in *SH2D1A* (Xq25) encoding signaling lymphocyte activation molecule-associated protein (SAP). Defective SAP induces serious immunological complications including impaired 2B4-mediated cytotoxicity of T or NK cells against EBV-infected cells, vigorous expansion of CD8+ T cells by a failure of T cell reactivation-induced cell death, and defects in the development of NKT cells (25, 26). XLP type 2-induced HLH is pathogenetically different from other genetic HLH, because cytotoxic lymphocyte-mediated cytotoxicity is apparently normal in patients with XLP type 2, which is caused by mutations of *XIAP/BIRC4* (27, 28). Instead, defective expression of XIAP increases a susceptibility of lymphocytes to apoptosis in response to CD95 and tumor necrosis factor receptor–related apoptosis-inducing ligand receptor stimulation, and induces defective NOD2 signaling with dysregulation of inflammasome function (27, 29, 30). Due to normal cytotoxicity, the development of HLH in these patients seems to have a less strong association with EBV, compared to patients with XLP type 1.

## Chronic Active EBV Infection of T- and NK- Cell Type, Systemic Form

CAEBV of systemic form is characterized by persistent clinical symptoms and signs including fever, hepatosplenomegaly, hepatitis, and lymphadenopathy after infectious mononucleosis (IM). Originally, when first described by Straus et al., the required duration of IM-like symptoms was more than 6 months to fulfill the criteria for CAEBV; however, the revised criteria require now only 3 months (3, 31, 32). The current diagnostic criteria are as follows: (1) IM-like symptoms persisting more than 3 months; (2) increased EBV DNA (>10^2.5^ copies/mg) in PB, (3) histological evidence of organ disease; and (4) demonstration of EBV RNA or viral protein in affected tissues (3). In addition, CAEBV should be diagnosed in patients without known immunodeficiency, malignancy or autoimmune disorders. Most cases have been reported in East Asia including Japan, South Korea, China, and Taiwan (33–36). Few reports come from Latin America. It appears to occur rarely in Western and African populations (37). CAEBV arises predominantly in pediatric and adolescent patients. If it develops in adults, it shows a more aggressive clinical course (38). No sex predilection is present.

### Clinical Features

The typical IM-like manifestations including persistent fever, hepatosplenomegaly and lymphadenopathy are present in about half of the patients. Some patients with CAEBV may have variable and non-specific symptoms according to the organs affected by EBV-induced inflammation, which often causes the diagnosis to be delayed or misdiagnosed. Other relatively common symptoms and signs are severe mosquito bite allergy (33%), skin rash (26%), HV-like eruptions (10%), diarrhea (6%), and uveitis (5%) (39). Pancytopenia and liver dysfunction are common. High titers of anti-VCA IgG and anti-early antigen IgG are found in almost all patients, and IgA antibodies against VCA and early antigen are frequently revealed. Increased copies of EBV DNA are also identified in all patients with CAEBV (39, 40).

The clinical course is variable. Some patients show an indolent clinical course and remain stable over a long period, while other patients progressively deteriorate with serious complications including hemophagocytic syndrome (24%), disseminated intravascular coagulation (16%), hepatic failure (15%), digestive tract ulcer/perforation (11%), coronary artery aneurysm (9%), CNS involvement (9%), interstitial pneumonia (5%), and myocarditis (7%) (4, 33, 39). The variability of clinical behavior generally depends on the viral load of EBV DNA and the host immunity. The prognosis is associated with the predominant cell type infected with EBV. Patients with CAEBV of T-cell type shows significantly poorer survival rate than those with CAEBV of NK-cell type (probability of 5-year survival, 0.59 in CAEBV of T-cell type vs. 0.87 in CAEBV of NK-cell type) (39). Furthermore, both groups tend to have different clinical presentations. Patients with T-cell infection are more likely to show severe systemic symptoms, high titers of EBV specific antibodies and aggressive clinical behavior, whereas those with NK-cell infection commonly have mild systemic symptoms, high concentrations of IgE, relatively lower levels of EBV-specific antibody and skin lesions including rash and hypersensitivity to mosquito bites, probably reflecting the more favorable clinical course of this type (4, 39, 41). However, CAEBV of NK-type (23.1%) might eventually evolve into aggressive NK-cell leukemia or extranodal NK/T cell lymphoma, nasal type (39).

### Morphology and Immunophenotypical Findings

The biopsy of affected tissues in CAEBV is characterized by reactive inflammatory changes with no histological evidence of a malignant lymphoproliferation. Because microscopic findings are similar to nonspecific inflammation, pathological diagnosis can be very difficult and overlooked without careful attention to the clinical history. The detection of EBV infection in the infiltrated lymphoid cells using EBER ISH is necessary for tissue confirmation, and very useful especially in patients with no clinical suspicion of having CAEBV, due to nonspecific and unusual presentations. Histological findings of LN include follicular hyperplasia, paracortical hyperplasia, focal necrosis, and small epithelioid granulomas (3). A polymorphic infiltrate can be noted occasionally in interfollicular areas. Atrophy of the white pulp and congestion of the red pulp are seen in the spleen. In liver biopsies, infiltration of small lymphocytes is shown in portal areas or sinusoids like in viral hepatitis. The BM appears to be microscopically normal, except cases with accompanying HLH. According to the affected organs, CAEBV can mimic interstitial pneumonia in lung biopsies, myocarditis in heart biopsies and dermatitis in skin biopsies. EBV-infected cells show either a T-cell immunophenotype (59%) or a NK-cell immunophetype (41%), and rarely EBV-infection in both T and NK cells (3%). T-cells are mostly CD4+ (21%), with a minority being either CD8+ (8%), or γδ T-cell type (5%). Ill-defined T-cell phenotypes occur in 25% of the cases. CAEBV of B-cell type occurs rarely (2–3%) and mainly in Western population (42).

### Pathogenesis and Molecular Features

The pathogenesis remains unknown. Although CAEBV develops in immunocompetent hosts by definition, some patients have impaired activity in EBV-specific cytotoxic T cells (43, 44). Strong racial differences in susceptibility to CAEBV suggest that its occurrence is influenced by genetic polymorphisms in host immune response-related genes (34, 45). The viral latency pattern of CAEBV indicates latency type 2, because EBV-infected T or NK cells express only a few EBV-related genes including EBNA1, LMP1 and LMP2A (41, 44). EBV is mononclonal in 84%, oligoclonal in 11%, and polyclonal in 5% of the reported cases (4). The rearrangement pattern of the TCR genes seems to be monoclonal in about 50 % of the cases (4). Therefore, monoclonality of EBV-infected cells does not warrant a diagnosis of malignant lymphoma in EBV-associated T and NK-cell LPDs. Chromosomal abnormalities have been noted in a minority of cases (4). A clinicopathological classification of EBV-associated T and NK-cell LPDs has been proposed according to morphological evaluation and clonality results, which consists of category A1 (polymorphic, polyclonal LPD), category A2 (polymorphic, monoclonal LPD), category A3 (monomorphic, monoclonal LPD), and category B (monomorphic, monoclonal LPD with fulminant course) (46). Categories A1–A3 represents a continuous spectrum of CAEBV and its evolution to overt lymphoma. Category B corresponds to systemic EBV-positive T-cell lymphoma of childhood in the 2016 revised WHO classification (3).

## Hydroa Vacciniforme-Like Lymphoproliferative Disease

HV-like LPD is one of the cutaneous forms of CAEBV. HV-like LPD is considered as a chronic EBV+ LPD that affects mainly children, but it carries a risk of progression to clinically overt malignant lymphoma. HV was initially described in Western populations as a benign photodermatosis that presents with recurrent self-limited vesiculopapular eruption, which progresses to crusts with rupture and heals with vacciniform scarring (47). Skin lesions mimicking classic HV were identified with some different features including no association with photosensitivity in children and young adolescents from Asia and Latin America (48–51). These lesions were shown to be associated with EBV infection and frequently contained monoclonal TCR gene rearrangements in subsequent studies (49, 50, 52). Reflecting these results, HV-like lymphoma was included in the 2008 WHO classification as a new type of EBV-positive cutaneous T-cell lymphoma of childhood. However, because this term does not represent the diverse clinical spectrum from classic self-limited HV to HV-like lymphoma, the term HV-like lymphoma was changed to HV-like LPD in the 2016 revised WHO classification (3).

### Clinical Features

HV-like LPD shows variable clinical presentations and behaviors (4, 53, 54). HV-like LPD was previously classified into the classic type and severe type, representing the two extremes of the clinical presentation. Patients with the classic HV have light-induced vesiculopapules in sun-exposed skin area including the face and arm without systemic symptoms, whereas the severe form is characterized by the complicated vesicles that progress to large skin ulcers and occasionally leave severe scarring with disfigurement in sun-exposed and unexposed areas, with frequent systemic symptoms such as fever, lymphadenopathy, and hepatosplenomegaly (55). Some mild cases are cured after photoprotection, but the majority of cases usually shows a very indolent clinical course and has multiple recurrences and remissions that may finally develop into a more severe disease including EBV-positive T or NK-cell lymphoma of skin. A seasonal variation is present with increased occurrences of episodes in spring and summer.

### Morphology and Immunophenotypical Findings

**Figure 1:**
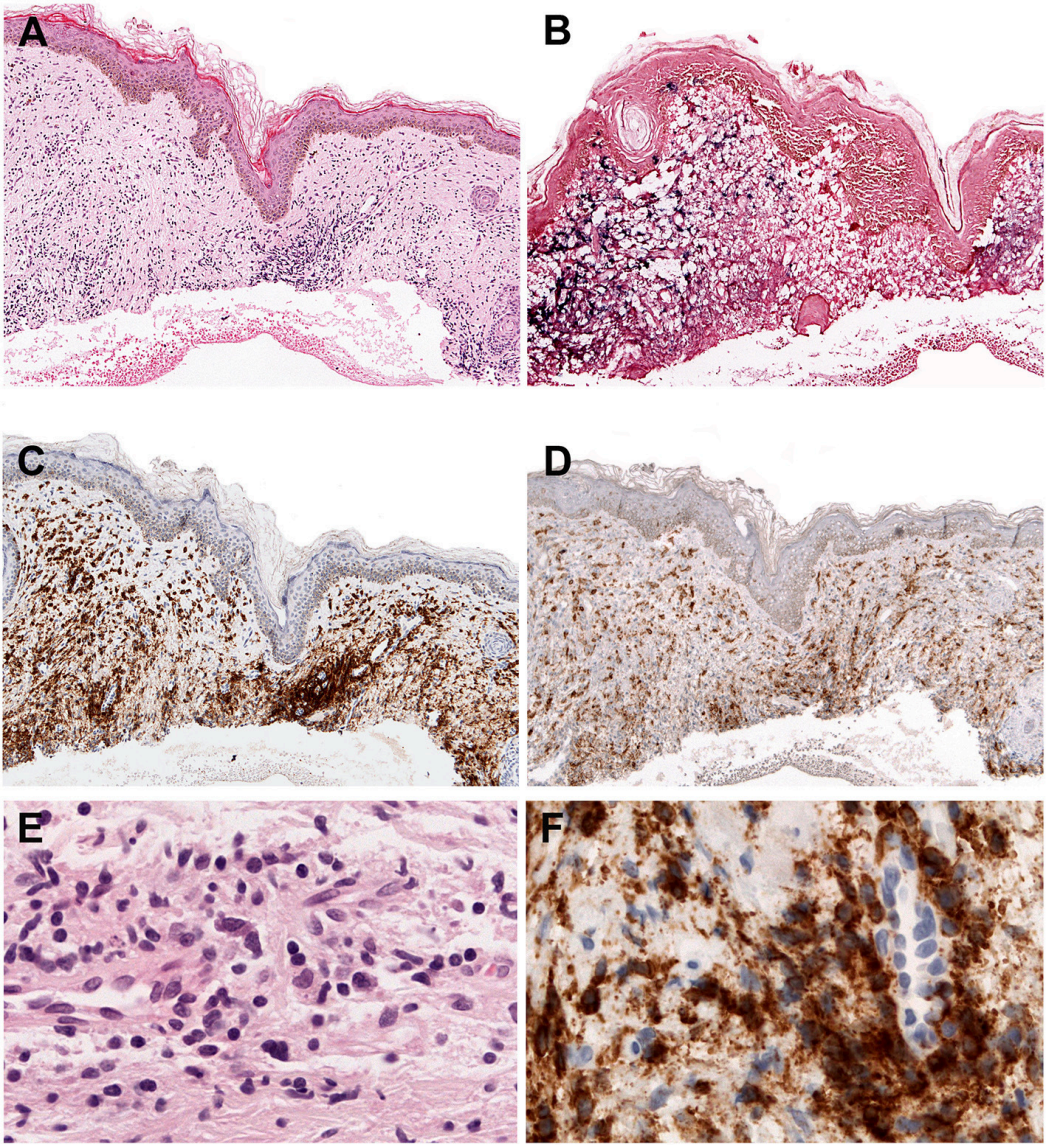
Hydroa vacciniforme-like lymphoproliferative disorder. **(A)** A skin biopsy with a subtle dermal infiltrate surrounding adnexae and blood vessels (H&E, 25x); **(B)** The lymphoid cells are EBV positive, as demonstrated by *in situ* hybridization for EBV-encoded small RNA (EBER) (*in situ* hybridization, 25x); **(C)** CD8 is positive in the majority of the infiltrating cells (immunohistochemistry, 25x); **(D)** The infiltrating cells are negative for CD4. CD4 highlights the abundant histiocytes (immunohistochemistry, 25x); **(E)** The infiltrating cells are predominantly small, without atypia (H&E, 400×); **(F)** The infiltrating cells are surrounding a blood vessel highlighted by CD8 stain (immunohistochemistry, 400×).

Intraepidermal spongiotic vesicles are characteristically formed by epidermal reticular degeneration. However, in some case only a very subtle lymphoid infiltration might be observed (Figures 1A–F). These lymphoid infiltrates are predominantly present in the dermis with the frequent extension to the subcutaneous tissue, and often exhibit an angiocentric and periadnexal involvement with the pattern of septal or lobular panniculitis. The infiltrating cells are usually bland-looking small lymphocytes with no or mild atypia, and highlighted by EBV positivity (Figures 1A,B,E). These cells are CD8+ cytotoxic T cells in the majority of cases (Figures 1C,D,F), followed by CD56+ NK cells (about 30%) (4, 56, 57). γδ T cells are found in a few cases in immunophenotyping of skin-infiltrating T cells, whereas flow cytometry of PB shows clonal expansion of γδ T cells in most cases (56, 58, 59). CD30 is frequently positive in the lymphoid infiltrates, and LMP1 is mostly negative (56).

### Pathogenesis and Molecular Features

The pathogenesis is unknown. The geographic and racial distribution pattern suggests that genetic predisposition related to a defective immune response to EBV may contribute to the susceptibility to HV-like LPD, like in other EBV-associated T, and NK-cell LPDs. Monoclonal rearrangement of the TCR genes is found in almost all cases with T-cell immunophenotype (4, 56). EBV-infected cells can be detected by EBER ISH. The number of EBV-positive cells is variable among cases, and there are only a small number of EBV-positive cells in some cases. Pathological parameters including T cell clonality or the quantity of EBV-positive cells are generally not associated with the clinical behavior or related to the risk to develop a systemic lymphoma (3). In contrast to LMP1 negativity shown by immunohistochemistry in tissues, LMP1 expression is mostly identified by PCR in PB, indicating an EBV latency type 2 (60).

## Severe Mosquito Bite Allergy

Severe mosquito bite allergy is another cutaneous form of CAEBV. Severe mosquito bite allergy is an EBV+ NK-cell LPD involving skin and characterized by an exaggerated allergic reaction to mosquito bites. Patients with severe mosquito bite allergy may be complicated by HLH, HV-like LPD, or systemic CAEBV in the prolonged clinical course. Furthermore, they have a higher risk of developing overt NK/T-cell lymphoma or aggressive NK-cell leukemia.

Severe mosquito bite allergy is a rare disease, of which most have been described in Japan with a few cases from other East Asia (4, 61–65). It mainly affects children and young adolescents aged 0–18 years (mean onset age, 6.7 years) (66). No sex predilection is reported.

### Clinical Features

Hypersensitivity reactions after mosquito bites include localized cutaneous manifestations such as erythema, bullae, ulcers, and scar formation, and systemic findings including high fever, lymphadenopathy, liver abnormalities, and hepatosplenomegaly. Some patients experience similar hypersensitivity reactions at the injection site after vaccination (66). Most patients exhibit a high serum IgE level, high EBV load, and NK-cell lymphocytosis in the PB. After the hypersensitivity reaction resolves, the patients remain asymptomatic until the next mosquito bites.

### Morphology and Immunophenotypical Findings

Epidermal necrosis, ulceration and bullae formation are present in the mosquito bite lesion. Skin biopsies from these lesions show a dense infiltrate of lymphoid cells that may extend into the subcutaneous tissue. The lymphoid infiltrate has a polymorphous composition consisting of small lymphocytes, large atypical cells, and other reactive inflammatory cells including histiocytes and eosinophils. The overall appearances are similar to HV-like LPD.

The infiltrating lymphoid cells are immunophenotypically CD3ε+, CD56+ NK cells with the expression of cytotoxic molecules TIA1 and granzyme B. Reactive CD4+ or CD8+ T cells are also present in the infiltrates. EBV-positive cells are often positive for CD30, and rarely positive for LMP1.

### Pathogenesis and Molecular Features

The etiology remains unknown. Genetic predispositions and environmental factors may have an influence on the pathogenesis. Mosquito bites can induce the expression of LMP1 in NK cells through the proliferation of mosquito antigen-specific, CD4+ T cells, which are involved in the reactivation of latent EBV in NK cells (67–69). EBV positivity is found only in a percentage of the infiltrating NK cells. EBV is monoclonal in almost all cases by EBV terminal repeat analysis (4). Like HV-like LPD, LMP1 expression is seen in PB by PCR, thereby, indicating type 2 EBV latency.

## Systemic EBV-Positive T-Cell Lymphoma of Childhood

Systemic EBV-positive T-cell lymphoma of childhood is a rapidly progressive, fatal disease of children and young adults that is characterized by monoclonal expansion of EBV-positive T cells with an activated cytotoxic phenotype in tissues or PB. Historically, several different terms have been used to describe this disease, including fulminant EBV+ T-cell LPD of childhood, sporadic fatal IM, fulminant hemophagocytic syndrome in children, fatal EBV-associated hemophagocytic syndrome, and severe CAEBV. Systemic EBV-positive T-cell lymphoma is almost always accompanied by HLH, and shows a fulminant clinical course, rapidly progressing to multiple organ failure, sepsis, and finally death, within days to weeks. This disease was first incorporated as a LPD in the 2008 WHO classification; however, in the current 2016 revised WHO classification, it has been renamed as systemic EBV-positive T-cell lymphoma of childhood, reflecting its clinical severity. Systemic EBV-positive T-cell lymphoma of childhood occurs mainly in East Asia including Japan, Taiwan and China (33, 70–72). It also occurs in Latin America but it is rare in Western populations (73).

### Clinical Features

Systemic EBV-positive T-cell lymphoma of childhood usually occurs shortly after acute primary EBV infection in previously healthy children or adolescents. Patients present with severe systemic findings such as fever, hepatosplenomegaly, pancytopenia, coagulopathy, and abnormal liver function, within days to weeks after IM symptoms due to primary EBV infection. Lymphadenopathy is occasionally seen. The patients are usually complicated by HLH, sepsis, and multiorgan dysfunction, ultimately leading to death within days to weeks.

In serological tests for EBV, anti-VCA IgM is often absent or barely detectable in the majority of patients, whereas IgG antibodies against VCA are positive. These abnormal results may be misleading and contribute to the delay in diagnosis, considering that they do not indicate acute or active EBV infection (74, 75).

### Morphology and Immunophenotypical Findings

**Figure 2:**
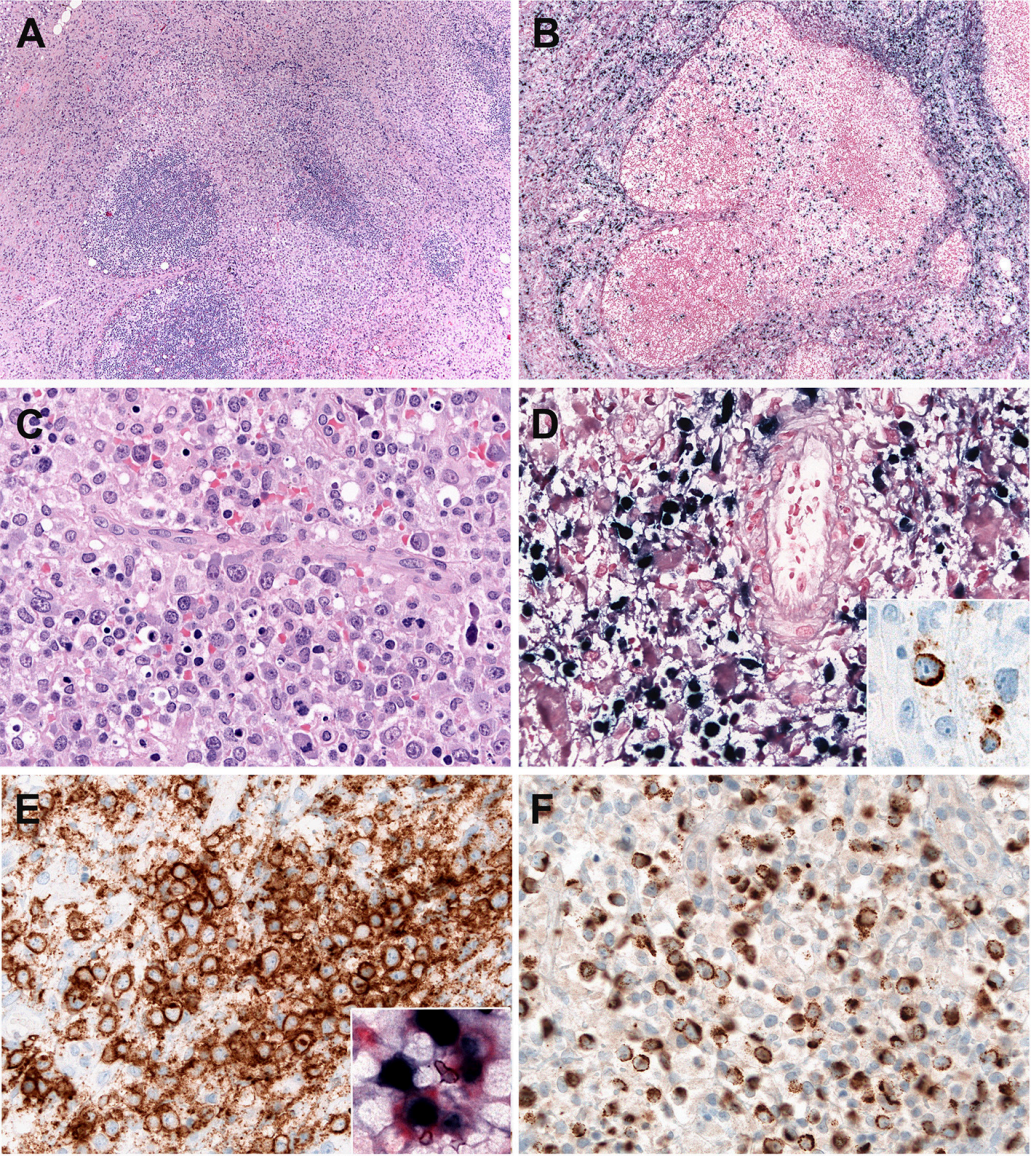
Systemic Epstein-Bar virus (EBV)-positive T-cell lymphoma of childhood. **(A)** Lymph node with partial preservation of the architecture with depleted germinal centers and expansion of the interfollicular area (H&E, 50×); **(B)** Many of the lymphoid cells in the interfollicular area are EBV-positive, as demonstrated by *in situ* hybridization for EBV-encoded small RNA (EBER) (*in-situ* hybridization 100×); **(C)** The neoplastic cells are mostly medium to large-sized cells with irregular nuclei. Note the presence of apoptosis (H&E, 400×); **(D)** Many cells are EBER positive (*in-situ* hybridization, 400×). LMP1 is positive indicating an EBV latency type 2 (immunohistochemistry, insert, 400×); **(E)** The neoplastic cells are CD8 positive (immunohistochemistry, 400×). Double stainings show that the CD8-positive cells (red) are EBER-positive (Black) (Immunohistochemistry and in situ hybridization, insert, 400×) **(F)** TIA1 is positive in the infiltrating cells (immunostaining, 400×).

Systemic EBV-positive T-cell lymphoma of childhood is histologically characterized by increased infiltration of small lymphoid cells with histiocytic hyperplasia and striking hemophagocytosis in the BM, spleen, and liver. Tumor cells have no or minimal cytological atypia in most cases and may not be distinguishable from normal lymphocytes. However, some cases show atypical lymphoid infiltrates composed of pleomorphic medium to large-sized cells with frequent mitosis (72) (Figure 2). The liver reveals mild to marked infiltration of small lymphocytes in portal and sinusoidal area with cholestasis, steatosis, and focal necrosis (73). The spleen shows the depletion of white pulp with prominent sinusoidal and nodular lymphoid infiltrates. The LNs are usually unremarkable with preserved architecture. Early histological findings of lymph nodes include the depletion of B-cell areas and the expansion of paracortical/interfollicular areas that is infiltrated by a polymorphous population of lymphoid cells consisting of small to medium-sized lymphocytes and large atypical lymphocytes with irregular nuclei (Figures 2A–F). The LNs appear to be more depleted along the disease progression. The infiltrating lymphoid cells are mostly EBV+, CD8+ cytotoxic T cells that express CD2, CD3, TIA-1, and granzyme B, and lack CD56 (71, 73). In contrast, cases associated with CAEBV show CD4+ immunophenotype (73). Rare cases exhibit EBV-positive tumor cells with co- expression of CD4 and CD8 (73). LMP1 is usually negative by immunohistochemistry. EBNA2 is always negative. The confirmation of EBV infection by EBV ISH is very useful for diagnosis.

### Pathogenesis and Molecular Features

The etiology of systemic EBV-positive T-cell lymphoma of childhood is unknown. However, its association with primary EBV infection and strong racial predisposition suggest a genetic defect in the host immune response to EBV. The infiltrating T cells show monoclonal rearrangements of the TCR genes. EBV is present in a clonal episomal form in all cases (33, 70, 72, 73, 76). Because systemic T-cell lymphoma of childhood have some clinicopathological characteristics overlapping with EBV-associated HLH, the distinction of both diseases is sometimes difficult. In a literature review of systemic T-cell lymphoma and EBV-associated HLH, the patients with chromosomal aberrations are 100% fatal, whereas cases with evidence for T-cell clonality are fatal in 62% (77). Therefore, karyotypic abnormalities can be more helpful to distinguish systemic T-cell lymphoma of childhood from EBV-associated HLH in ambiguous cases, compared to T cell clonality, which is demonstrated in about half of EBV-associated HLH cases.

## Aggressive NK-Cell Leukemia

Aggressive NK-cell leukemia is a very rare, fatal disease characterized by systemic neoplastic proliferations of NK cells in PB and BM and a strong association with EBV. However, leukemic NK cells are variably present, and sometimes can be sparse in the PB and BM. It was originally designated as aggressive NK-cell leukemia/lymphoma to emphasize the variable clinical manifestations, because there are some cases without leukemic phase, presenting with hepatosplenomegaly and peripheral lymphadenopathy (78, 79). To permit a clear distinction, and avoid confusion between this disease and extranodal NK/T-cell lymphoma, nasal type, the term aggressive NK-cell leukemia has been chosen in the WHO classification. Extranodal NK/T-cell lymphoma with systemic involvement at multiple sites has some clinicopathological features similar to those of aggressive NK-cell leukemia, and the distinction between the two diseases may be difficult. Aggressive NK-cell leukemia has been reported mainly in Asians (80). It occurs mostly in young to middle-aged adults with no definite sex predilection.

### Clinical Features

Clinical presentations include high fever, general malaise, hepatosplenomegaly, hepatic failure, and pancytopenia. Variable numbers of leukemic NK cells are present in the PB, ranging from <5% to >80% of all leukocytes (75). Laboratory tests show high levels of serum lactate dehydrogenase (LDH) levels and circulating FAS ligand (FASL) (81). Lymphadenopathy is occasionally seen, and skin lesions are uncommon. This disease is frequently complicated by HLH and coagulopathy, and shows a fulminant clinical course with multiple organ failure (75). The overall prognosis is very poor with a median survival <2 months (82). Some cases develop in the setting of CAEBV infection of NK cell type, or evolve from extranodal NK/T-cell lymphoma or chronic LPD of NK cells (4, 33, 83–86). Aggressive NK-cell leukemia shares some clinicopathological features with systemic EBV-positive T-cell lymphoma of childhood, but the immunophenotype of the neoplastic cells is basically different between these two disease entities (CD56+ NK cells vs. CD56- T cells).

### Morphology and Immunophenotypical Findings

**Figure 3:**
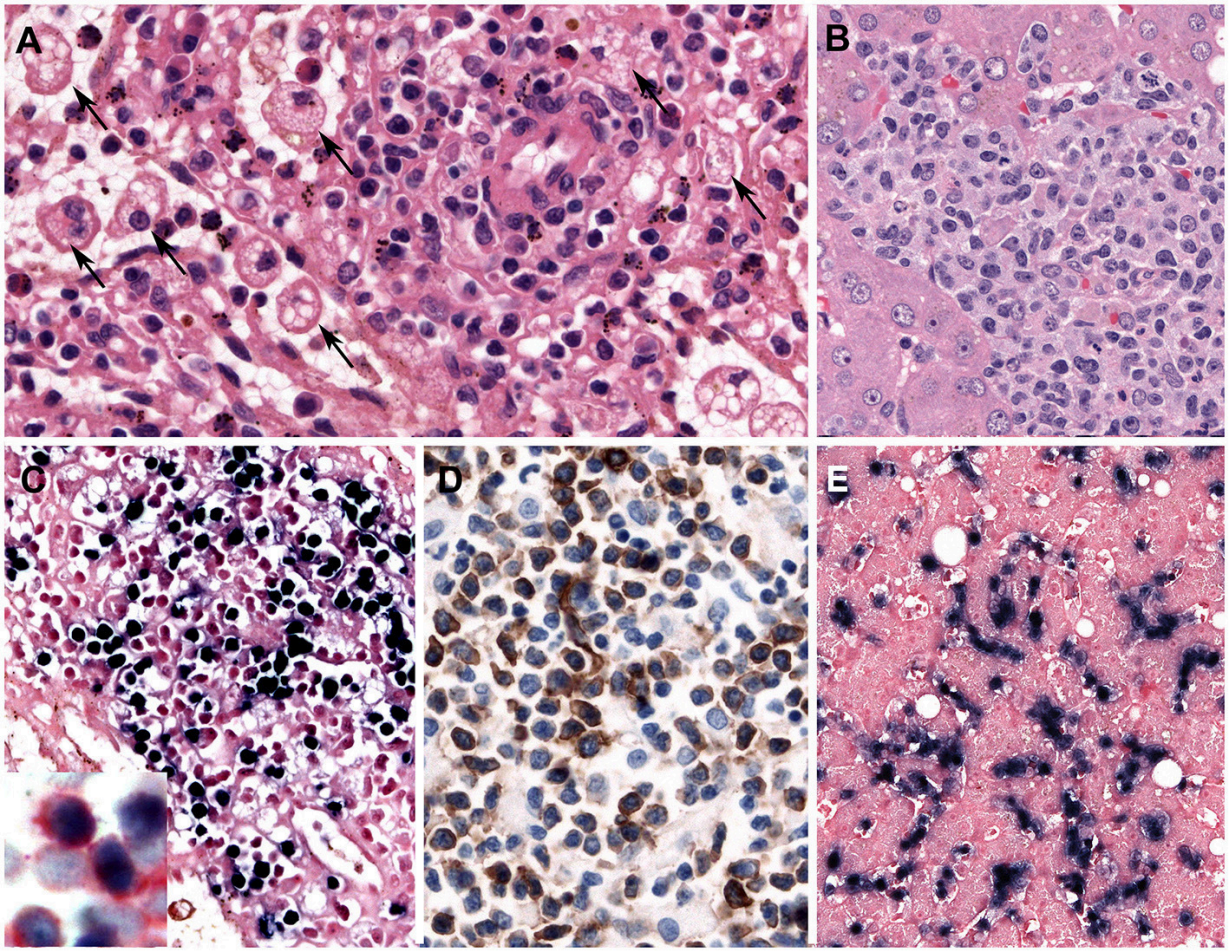
Aggressive NK-cell leukemia. **(A)** The spleen shows a scant atypical lymphoid infiltrate of small cells with bland cytology surrounding blood vessels. Note the striking erythrophagocytosis (arrows) (H&E, 400×); **(B)** The liver shows an atypical infiltrate in the sinusoids composed of medium-sized cells with irregular nuclei and pale cytoplasm; **(C)** Neoplastic cells in the spleen are stained positively with EBER (*in-situ* hybridization 400×). Insert shows double staining of CD56 (red) and EBER (black) demonstrating that the NK cells are infected by EBV (Immunohistochemistry and *in situ* hybridization 400×); and **(D)** The infiltrating cells are CD56 positive (immunohistochemistry 400×); **(E)** The neoplastic cells in the liver are EBER positive. Note the intrasinusoidal infiltration characteristic of the disease (*in-situ* hybridization, 400×).

Leukemic NK cells have a broad cytological spectrum ranging from normal large granular lymphocytes to atypical lymphocytes showing nuclear enlargement, irregular nuclear contours, open chromatin, or conspicuous nucleoli. The cytoplasm is pale or lightly basophilic, and relatively abundant with fine or coarse azurophilic granules. In the BM biopsy, the extent of neoplastic NK cells varies from extensive to focal, subtle infiltration. Some cases show minimal involvement of the BM, indistinguishable from normal BM in conventional H&E stain. Increased infiltration of histiocytes is observed with hemophagocytosis in the BM. The liver, spleen, and LNs show varying degrees of tumor cell infiltration, displaying massive, patchy, or subtle involvement, like the BM (Figures 3A–E). The immunophenotype of the tumor cells is that of a mature CD56+ NK-cell with positivity for CD2, CD3ε, and cytotoxic granules TIA1 and granzyme B, and negative for surface CD3 and CD5. Aggressive NK-cell leukemia frequently expresses CD16 (in 75%), which is different from CD16-negative extranodal NK/T-cell lymphoma (82). The neoplastic cells express FASL, but usually lack CD57. The early diagnosis of aggressive NK-cell leukemia can be difficult due to unusual pathological findings including lack of lymphocytosis in PB, interstitial infiltration pattern in BM, rarely EBER negativity and aberrant immunophenotype such as CD3 negativity by immunohistochemistry (87).

### Pathogenesis and Molecular Features

Although the etiology remains unknown, the strong association with EBV has been suggested to be central in pathogenesis. EBV infection has been reported in 85–100% of cases (88–90). EBV exists in clonal episomal form. However, some cases of EBV-negative aggressive NK-cell leukemia have been described, and show the clinicopathological features similar to those of EBV-positive cases, except for the fact that EBV-negative cases tend to occur in older patients with no obvious racial predilection (87, 91). Some of the EBV-negative cases may evolve from chronic LPD of NK cells (82, 91, 92). The genetic comparison of aggressive NK-cell leukemia and extranodal NK/T-cell lymphoma show some significant differences. Aggressive NK-cell leukemia shows gains of 1q23.1–q24.2 and 1q31.3–q44 and losses of 7p15.1–p22.3 and 17p13.1 more frequently than extranodal NK/T-cell lymphoma (93). A previous study reported that EBV-negative aggressive NK-cell leukemia was negative for JAK-STAT pathway-associated gene mutations, known as recurrently mutated in extranodal NK/T-cell lymphoma, suggesting different molecular pathogenesis between these two diseases (87). However, because the mutations of the JAK-STAT pathway-associated genes have been also reported in EBV-positive aggressive NK-cell leukemia (94), further investigations are warranted to reveal the mutational landscape of the NK-cell malignancies.

## Extranodal NK/T-Cell Lymphoma, Nasal Type

Extranodal NK/T-cell lymphoma, nasal type, is an EBV-positive aggressive lymphoma characterized by prominent necrosis, angioinvasion, and cytotoxic phenotype. It is derived from NK cells and uncommonly cytotoxic T cells. Extranodal NK/T-cell lymphoma was called “lethal midline granuloma” in the past, because of the destruction of midline facial structures due to vascular damage and subsequent ischemic necrosis. Extranodal NK/T-cell lymphoma, nasal type, occurs commonly in East Asians and the Native Americans in Central and South America, but rarely in Western populations (95). It accounts for ~6–8% of all lymphomas in East Asia and some Latin American countries, but <1% in Western populations (96–100). It affects males more commonly than females. It occurs frequently in middle-aged adults.

### Clinical Features

**Figure 4:**
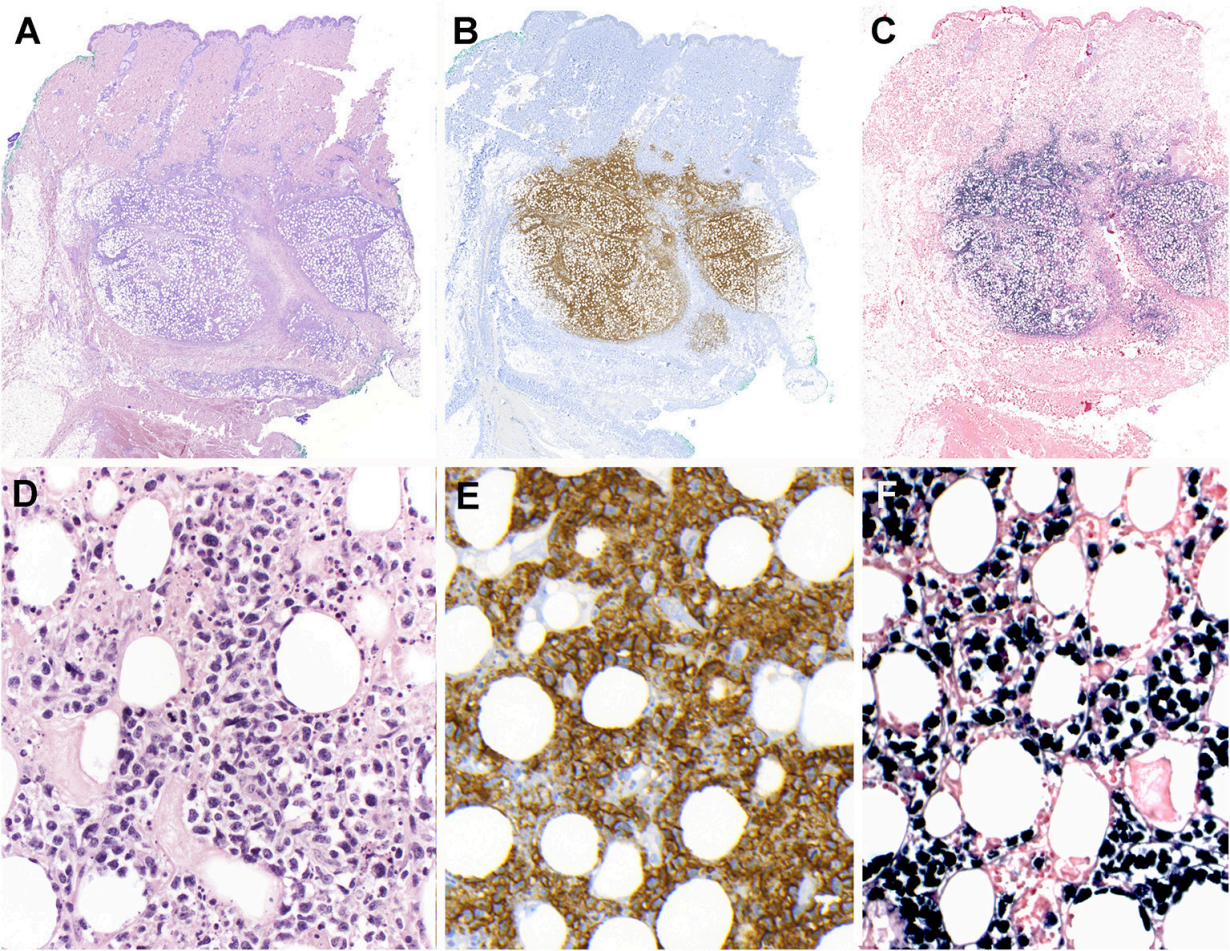
Extranodal, NK/T-cell lymphoma, nasal type in the skin. **(A)** Panoramic view of a skin biopsy shows a partially circumscribed nodule located in the subcutaneous tissue (H&E, scanned slide); **(B)** The tumor cells are CD56 positive (immunohistochemistry, scanned slide). **(C)** The lymphoid cells are positive for EBV-encoded small RNA *in situ* hybridization (EBER) (*in situ* hybridization) **(D)** The infiltrate is composed of large atypical cells with irregular nuclei. The tumor cells surround the adipocytes revealing a “lace-like pattern” mimicking panniculitis-like T-cell lymphoma. Numerous apoptotic bodies are observed (H&E, 400×); **(E,F)** Higher magnification demonstrates that the neoplastic cells are positive for CD56 and EBER, (immunohistochemistry and *in situ* hybridization 400×).

Extranodal NK/T-cell lymphoma arises from extranodal sites in almost all cases, most commonly involving upper aerodigestive tract (UAT) including nasal cavity, paranasal sinuses, nasopharynx, oropharynx, oral cavity, and palates. It also occurs in non-UAT sites including skin, soft tissue, gastrointestinal tract, testis, lung, and CNS. Nasal NK/T-cell lymphoma is defined as a primary tumor involving the nasal and nasopharyngeal region, regardless of dissemination to other sites. Patients with nasal NK/T-cell lymphoma present initially with nonspecific localized symptoms including nasal obstruction, purulent nasal discharge, and epistaxis. In later stages, nasal NK/T-cell lymphoma can extend to adjacent UAT tissues, or cause extensive necrotic lesions in the midline facial area. The BM is infrequently involved. Some patients may be complicated by HLH and extranasal dissemination to various non-UAT sites. The prognosis of nasal NK/T-cell lymphoma has been reported to be poor with the overall survival rate of 30–40%, but has recently improved with the introduction of new chemotherapy regimens such as L-asparaginase-based chemotherapy (95, 101). Extranasal NK/T-cell lymphoma indicates a primary tumor in a non-UAT site at first clinical presentation, and occurs in ~20–30% of cases (Figure 4) (100, 102). These lymphomas usually present with advanced stage at diagnosis, multiple sites of involvement, elevated LDH levels, and poor performance status. They are often refractory to treatment and show an inferior prognosis compared to nasal NK/T-cell lymphoma (101). Skin lesions commonly present as nodular ulcerative lesions. Gastrointestinal involvement frequently results in ulcer, bleeding, or perforation. Lymph nodes can be secondarily involved by dissemination. As some extranasal cases may harbor occult nasal lymphoma, it is important to inspect the UAT regions carefully.

### Morphology and Immunophenotypical Findings

**Figure 5:**
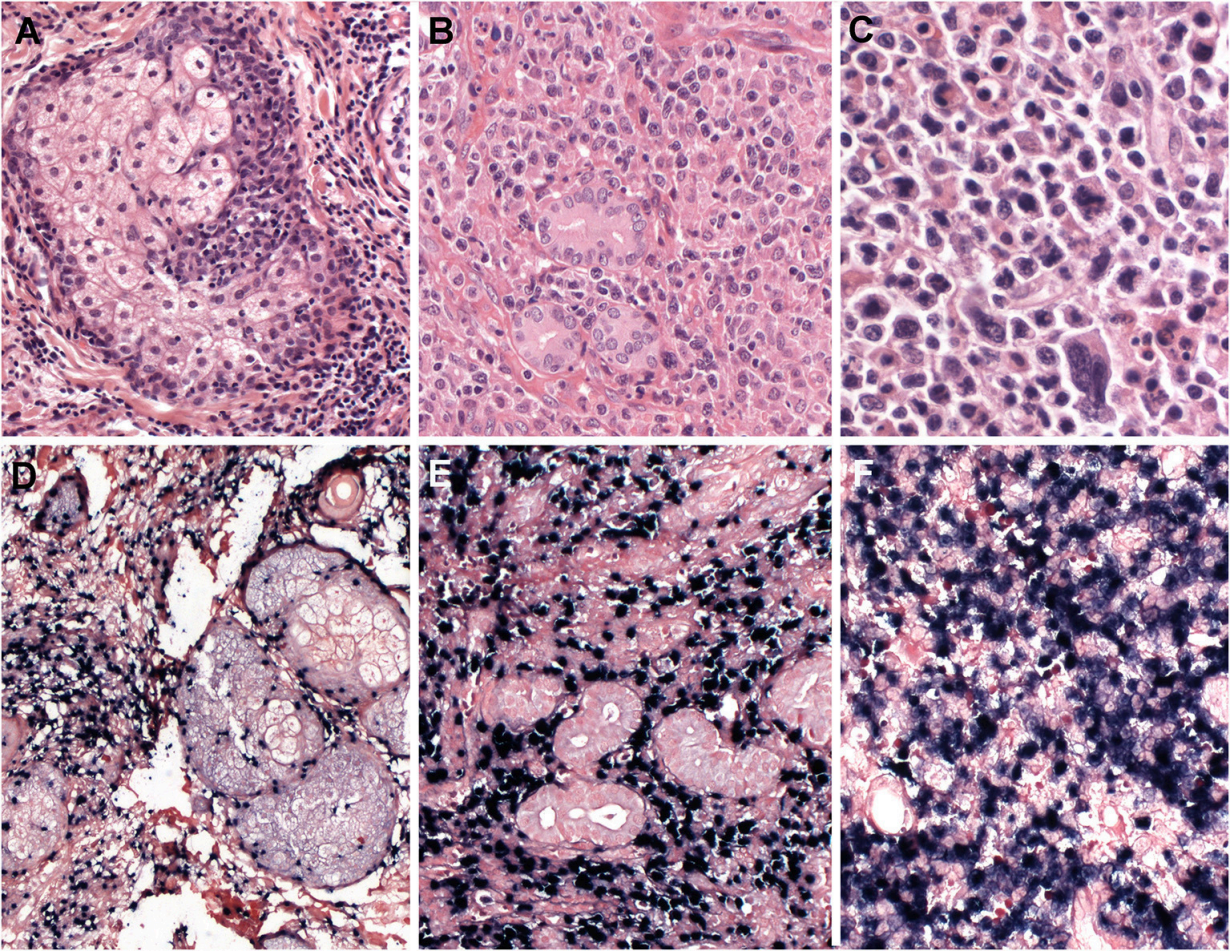
Extranodal, NK/T-cell lymphoma, nasal type. **(A–C)** Nasal biopsies displaying the morphological spectrum of ENKTCL. **(A)** Infiltrate of small lymphoid cells with bland cytology surrounding the sebaceous gland, mimicking a reactive lesion (H&E, 400×);**(B)** Dense lymphoid infiltrate of intermediate-sized cells, showing nuclear irregularity and pale to clear cytoplasm (H&E, 400×); **(C)** Atypical cell infiltrate, of pleomorphic large cells admixed with intermediate-sized cells (H&E, 400×); **(D–F)** Lymphoma cells are positive for EBV-encoded small RNA *in situ* hybridization (EBER, 400×).

Involvement of mucosal sites frequently presents with extensive ulceration. Histological findings show diffuse infiltration of atypical lymphoid cells with angiocentricity and angiodestruction, leading to vascular obstruction and the consequent ischemic, coagulative necrosis. Involvement of non-mucosal sites also shows similar morphological changes. Tumor cells have a broad cytological spectrum, ranging from bland-looking small lymphocytes to large pleomorphic cells (Figures 4A–F). Most cases show a relatively monotonous population of medium-sized cells or a polymorphous pattern composed of small and large cells. Variable amounts of reactive inflammatory cells are admixed with tumor cells, mimicking an inflammatory lesion. Tumor cells have often irregularly folded nuclei with indistinct nucleoli and moderate amount of cytoplasm. Mitotic figures are easily identified. The most common immunophenotype is CD3ε+, CD56+, CD2+ and cytotoxic molecules (granzyme B, perforin and TIA1), but lacks surface CD3, CD4 and CD5 (Figures 4, 5). Tumor cells often express CD25, FAS, FASL, and HLA-DR. CD30 expression is identified in about 30–40% of cases (103–106). The minority of cases shows a CD3+, CD56- cytotoxic T-cell phenotype, expressing CD8, CD5,TCR (γδ or αβ type), and cytotoxic molecules. These cases account for 15–20% of all cases and represent a real T-cell phenotype of the tumor cells (104, 107). There are no significant differences in the clinicopathological features between CD56+ and CD56- cases (88). EBV infection should be confirmed in virtually all cases to render a diagnosis of extranodal NK/T-cell lymphoma. Therefore, when CD3ε+, CD56+ nasal lymphomas do not show EBV positivity, other types of T-cell lymphoma should be considered in the diagnosis. Because of a strong association with EBV, the immune microenvironment has some prognostic implication. High quantity of tumor-infiltrating FOXP3+ regulatory T cells or PD-L1 expression on tumor cells independently predict better prognosis, suggesting that inhibitory immunomodulation might suppress lymphoma cells, as well as immune cells related with antitumor immune response (108, 109).

### Pathogenesis and Molecular Features

EBV has a pathogenetic role in the development of extranodal NK/T-cell lymphoma, nasal type. EBV exists in a clonal episomal form with EBV type 2 latency. Most patients are infected with EBV subtype A with some geographic variations (110–112). EBV has frequently a 30-base pair deletion in LMP1 gene, which may contribute to lymphomagenesis through decrease in immune recognition (113–115). The quantity of circulating EBV DNA reflects the tumor load and activity, because EBV DNA is released into the blood from apoptotic tumor cells. Elevated EBV DNA copies are correlated to adverse clinical parameters, poor response to treatment and inferior clinical outcomes (116, 117). Most cases show a germline configuration of TCR genes. Monoclonal rearrangements of the TCR genes are found in 10–40% of cases, which may be derived from cytotoxic T cells (100, 107, 118). In the gene expression profiling, extranodal NK/T-cell lymphomas cluster together, regardless of NK cell or γδ T-cell phenotype, and show patterns similar to non-hepatosplenic γδ T-cell lymphomas (119). The most frequent chromosomal aberrations in extranodal NK/T-cell lymphoma are deletion of chromosome 6q at q21–23 region, which contains some tumor suppressor gene such as *HACE1, PRMD1, FOXO3*, and *PTPRK* (120–122). This deletion is also commonly found in aggressive NK-cell leukemia, suggesting a genetic link between these two diseases. However, it is unknown whether this genetic alteration develops as primary pathogenetic event or secondary progression-related event (123, 124). Mutation analyses have shown that activating mutations of *JAK3* (5–35%), *STAT3* (6–27%) and *STAT5B* (2–6%) are commonly found, suggesting that the *JAK-STAT* pathway can be a therapeutic target (125–127). Other mutations include the RNA helicase *DDX3X*, the tumor suppressor gene *TP53*, the transcription corepressor BCOR, and genes involved in epigenetic pathways (*MLL2, ASXL3, ARID1A*, and *EP300*) (127, 128).

## Primary EBV-Positive Nodal T and NK-Cell Lymphoma

Primary EBV-positive nodal T and NK-cell lymphoma is a rare type of peripheral T-cell lymphoma that primarily involves the LNs without nasal or other extranodal site involvement. It has been included in the current 2016 WHO classification as a new provisional group within peripheral T-cell lymphoma, not otherwise specified (PTCL, NOS). It affects mainly elderly patients with a median age of 61 years, showing an older age distribution than extranodal NK/T-cell lymphoma (129). There is a male predilection (129, 130).

### Clinical Features

Most patients present with generalized lymphadenopathy. Extranodal involvement may be present in a limited number of sites, but the nasal cavity and adjacent structures should not be involved by definition. This disease shows an aggressive clinical course with adverse clinical features including advanced stage, systemic symptoms, and high International Prognostic Index scores. The prognosis is very poor with a median survival <4 months (129, 130).

### Morphology and Immunophenotypical Findings

**Figure 6:**
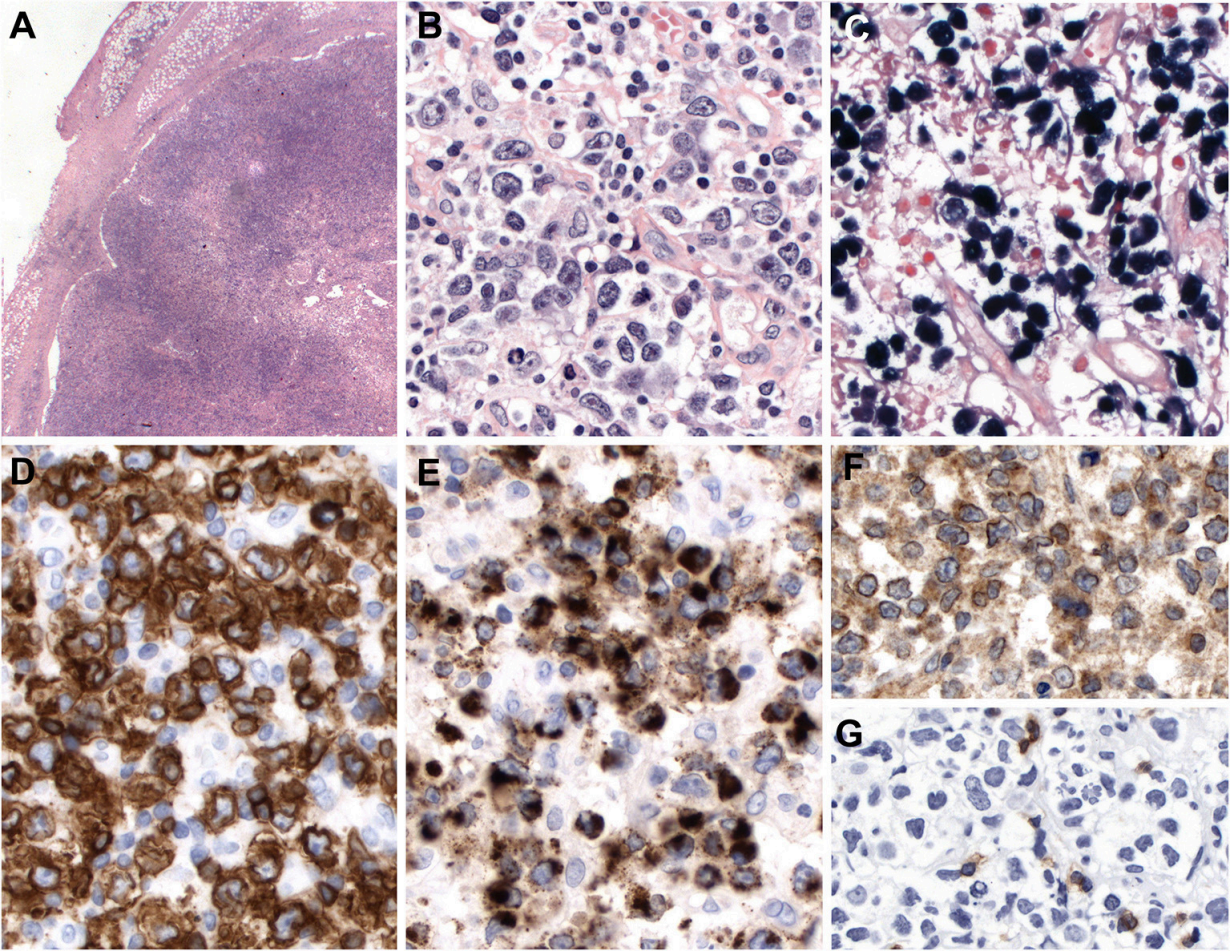
Primary EBV-positive nodal T and NK-cell lymphoma. **(A)** Lymph node with complete effacement of the architecture by a diffuse infiltrate that extends beyond the capsule and infiltrate the perinodal fat (H&E, 12,5×); **(B)** Neoplastic cells are large, pleomorphic with irregular nuclei and clear or pale cytoplasm (HE,400×); **(C–E)** The neoplastic cells EBER, CD56 and TIA-1 positive (*in-situ* hybridization and immunohistochemistry, 400×); **(F)** TCR-gamma immunostain demonstrates the gamma-delta derivation of the tumor cells (immunohistochemistry, 400×); **(G)** TCR alpha-beta (BetaF1) is negative in the tumor cells but positive in the reactive T cells (immunohistochemistry, 400×).

Most cases show relatively monomorphic proliferation of large atypical cells with centroblastic features or diffuse proliferation of pleomorphic cells composed of small, medium, to large atypical cells mimicking Hodgkin cells and Reed-Sternberg cells (Figure 6). Necrosis is occasionally seen with granulomatous or epithelioid histiocytic reactions. Angiodestructive pattern is rare. The immunophenotype of the tumor cells is mostly that of a cytotoxic T cell with expression of CD3, CD8, and cytotoxic molecules. Expression of CD56 (7.5–15%) or CD4 (15–20%) is rarely observed (129, 130). The majority of cases are of αβ T cell phenotype (46–64%), followed by TCR-silent T cells (negative for both TCRβF1 and TCRγ; 21–26%) and other T cells (130). Cases with NK-cell phenotype are found in 6.6–15%. CD30 expression is frequently expressed in TCR-silent cases, which raises the differential diagnosis with anaplastic large cell lymphoma (ALCL).

### Pathogenesis and Molecular Features

Monoclonal rearrangements of the TCR genes are found in most cases. EBV infection is diffusely detected with high density by EBER ISH, and LMP1 expression indicates EBV latency type 2. In a recent gene expression profiling and cytogenetic analysis, primary EBV-positive nodal T/NK-cell lymphoma showed a distinct molecular signature characterized by upregulation of PD-L1 and T-cell-related genes, including CD2 and CD8, and downregulation of CD56, compared to extranodal NK/T-cell lymphoma (131). A cytogenetic deletion frequently found is loss of chromosome 14q11.2 indicating loss of TCR loci as evidence for the T-cell origin of this lymphoma (131).

## Conclusion

EBV-associated T and NK-cell LPDs are a group of diseases including reactive LPDs, as well as overt hematolymphoid malignancies. Some categories have an indolent clinical course with pathological features mimicking a reactive disorder delaying the appropriate diagnosis and treatment. It is crucial to have good clinical information to render the correct diagnosis since some of these disorders have overlapping morphological features. It is recommended to perform EBER ISH in biopsies from extranodal sites with “atypical” infiltrations of T and NK cells regardless of the severity of the infiltration to confirm the presence of EBV infection. EBV+ T and NK cell LPDs frequently express CD30, which might be misleading raising the diagnosis of ALK- ALCL. New genetic studies suggest that the new provisional group of primary nodal T and NK cell lymphomas might be a distinct entity among the EBV+ LPDs.

## Author Contributions

WYK performed the literature review and wrote the manuscript. IM-M prepared all photographs and images. FF helped writing the manuscript. LQ-M supervised the work and helped writing the manuscript.

### Conflict of Interest Statement

The authors declare that the research was conducted in the absence of any commercial or financial relationships that could be construed as a potential conflict of interest.
